# An Evaluation of Web-Based Clinical Practice Guidelines for Managing Problems Associated with Cannabis Use

**DOI:** 10.2196/jmir.2319

**Published:** 2012-12-07

**Authors:** Melissa M Norberg, Michael W Turner, Sally E Rooke, Julia M Langton, Peter J Gates

**Affiliations:** ^1^University of New South WalesNational Cannabis Prevention and Information CentreRandwickAustralia; ^2^National Health ServiceAberdeenUnited Kingdom; ^3^University of SydneyFaculty of PharmacySydneyAustralia

**Keywords:** Cannabis, Marijuana Abuse, Addiction, Psychotherapy, Standards, Information Dissemination, Health Plan Implementation, Internet

## Abstract

**Background:**

Cannabis is the most widely used illicit substance, and multiple treatment options and avenues exist for managing its use. There has been an increase in the development of clinical practice guidelines (CPGs) to improve standards of care in this area, many of which are disseminated online. However, little is known about the quality and accessibility of these online CPGs.

**Objective:**

The purpose of study 1 was to determine the extent to which cannabis-related CPGs disseminated online adhere to established methodological standards. The purpose of study 2 was to determine if treatment providers are familiar with these guidelines and to assess their perceived quality of these guidelines.

**Methods:**

Study 1 involved a systematic search using the Google Scholar search engine and the National Drugs Sector Information Service (NDSIS) website of the Alcohol and Other Drugs Council of Australia (ADCA) to identify CPGs disseminated online. To be included in the current study, CPGs needed to be free of charge and provide guidance on psychological interventions for reducing cannabis use. Four trained reviewers independently assessed the quality of the 7 identified guidelines using the Appraisal of Guidelines for Research and Evaluation (AGREE II) tool. Study 2 assessed 166 Australian cannabis-use treatment providers’ (mean age = 45.47 years, SD 12.14) familiarity with and opinions of these 7 guidelines using an online survey. Treatment providers were recruited using online advertisements that directed volunteers to a link to complete the survey, which was posted online for 6 months (January to June 2012). Primary study outcomes included quality scores and rates of guideline familiarity, guideline use, and discovery methods.

**Results:**

Based on the AGREE II, the quality of CPGs varied considerably. Across different reporting domains, adherence to methodological standards ranged from 0% to 92%. Quality was lowest in the domains of rigor of development (50%), applicability (46%), and editorial independence (30%). Although examination of AGREE II domain scores demonstrated that the quality of the 7 guidelines could be divided into 3 categories (high quality, acceptable to low quality, and very low quality), review of treatment providers’ quality perceptions indicated all guidelines fell into 1 category (acceptable quality). Based on treatment providers’ familiarity with and usage rates of the CPGs, a combination of peer/colleagues, senior professionals, workshops, and Internet dissemination was deemed to be most effective for promoting cannabis use CPGs. Lack of time, guideline length, conflicts with theoretical orientation, and prior content knowledge were identified as barriers to guideline uptake.

**Conclusions:**

Developers of CPGs should improve their reporting of development processes, conflicts of interest, and CPGs’ applicability to practice, while remaining cognizant that long guidelines may deter implementation. Treatment providers need to be aware that the quality of cannabis-related CPGs varies substantially.

## Introduction

Clinical practice guidelines (CPGs) can facilitate appropriate clinical decision making and improve standards of care [1], but their effectiveness relies upon their quality [2]. Unfortunately, early research found that most CPGs published in the peer-reviewed medical literature, including those guidelines developed by specialty societies, were of poor quality [3,4]. Flawed guidelines may provide inaccurate scientific and clinical advice to treatment providers, thereby harming patients because of suboptimal or ineffective treatment delivery [2]. Therefore, the benefits of CPGs are contingent upon their development process. Although efforts have been made to improve the quality of peer-reviewed CPGs [5-7], many continue to lack rigorous development, editorial independence, and applicability to practice, or at least fail to adequately report on these issues [8-10]. The poor quality of CPGs may be perpetuated by the growing trend of publishing CPGs online that do not require peer review or the documentation of a systematic literature review unless they are indexed in a CPG database.

Although Internet dissemination is purported to increase accessibility by making CPGs freely available and by reducing publication delays associated with peer-reviewed journal submission, it may not have a corresponding effect on implementation [11,12]. A systematic review of dissemination strategies found that passive dissemination of educational material alone was not very effective for improving professional practice, but that the impact of educational material was enhanced when it was delivered through interactive educational meetings [13]. Other reviews have found that incorporating a combination of different activities is usually the most effective approach for getting health practitioners to change their behavior [14,15]. Based on these findings, the success of CPGs is not only dependent upon their quality, but it may also depend on which or how many dissemination strategies are used.

Treatment providers should choose which CPG to adopt based on a rigorous review process. Unfortunately, this may not be possible for those who do not have the training and/or time to scrutinize the methods by which guidelines were developed. Given that cannabis is the most frequently used illicit substance [16] and that multiple treatment options (eg, motivational enhancement therapy, cognitive behavior therapy, and family therapy) [17] and avenues (eg, inpatient, outpatient, rehabilitation, and primary care) [18] exist for managing cannabis use, there is a clear need for qualified individuals to evaluate the quality of cannabis-related CPGs, especially those disseminated online.

The purpose of study 1 was to demonstrate the extent to which cannabis-related CPGs adhere to established methodological standards using the validated Appraisal of Guidelines for Research and Evaluation (AGREE II) tool [19]. Its predecessor, the AGREE, is the most promising critical appraisal tool for CPGs and has been used to evaluate numerous CPGs across a variety of health issues [20]. The AGREE II was developed to improve upon the AGREE’s reliability and usability. The purpose of study 2 was to determine treatment providers’ familiarity with and views about the CPGs identified in study 1. Study findings will assist treatment providers in identifying high quality CPGs and assist guideline developers in improving their reporting and dissemination practices.

## Methods

### Study 1

#### Selection Criteria

To be included in the current study, CPGs needed to provide guidance on psychological interventions for reducing cannabis use. Interventions needed to target cannabis use broadly rather than one specific facet of reducing/ceasing use (eg, withdrawal). In addition, CPGs needed to be developed for professionals whose primary role is to provide counseling (ie, psychologists or counselors) and be available free of charge via the Internet. Further, CPGs were included only when the word *cannabis* (or a similar term) was in the title or in the table of contents (or similar summary list). Guidelines that primarily targeted professionals whose secondary role may include counseling (eg, nurses or general practitioners) or guidelines that were published in any language other than English were excluded. Client population was not an exclusion criteria.

#### Search Strategy

**Figure 1:**
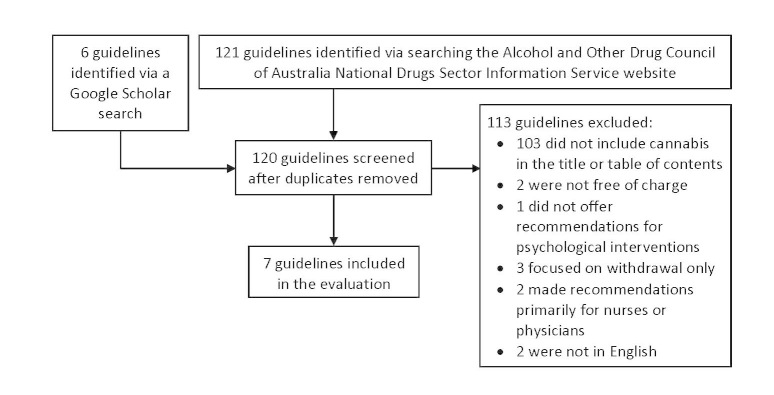
Selection of Web-based guidelines.

Two authors (MMN and SER) independently conducted a search of cannabis treatment guidelines using the Google Scholar search engine and the National Drugs Sector Information Service (NDSIS) website of the Alcohol and Other Drugs Council of Australia (ADCA), a service that provides direct and indirect access to guidelines through links [21]. The NDSIS contained 11 links to websites containing guidelines, but did not have a search facility. All websites accessed via links on this site were searched using the terms *marijuana*, *cannabis*, and *guidelines* if they had a search facility, otherwise links were followed to appropriate guidelines. Four websites contained eligible guidelines or contained links to other websites that had eligible guidelines: the Medical Observer [22], the National Guideline Clearinghouse [23], the National Institute for Health and Clinical Excellence website [24], and the Trip database [25]. The Medical Observer, the National Guidelines Clearinghouse, and the National Institute for Health and Clinical Excellence websites each contained 1 eligible CPG, whereas the Trip database contained 2 eligible CPGs for this study. In addition, the National Guideline Clearinghouse had a page with links to complementary websites. These links were explored and led to the discovery of 1 eligible guideline via the Guidelines International Network [26]. In total, 6 CPGs were sourced via NDSIS links to other websites following these methods. Next, Google Scholar was searched using the terms *guidelines*, *cannabis*, and *marijuana*. This returned 1290 hits, with titles revealing 6 potentially eligible CPGs. Only 1 of the 6 CPGs was eligible. Both authors agreed on the eligibility/ineligibility of each CPG (see Figure 1).

#### Quality Assessment

All 5 authors read the AGREE II manual and completed the online AGREE II training [19,27]. One author (MMN) selected 4 non-cannabis-related guidelines for pilot testing. Pilot testing allowed all authors to become familiar with the AGREE II assessment procedure and clarify any discrepancies in subjective quality ratings. The AGREE II appraisals were completed independently by 4 authors (the reviewers MWT, SER, JML, and PJG) and then reviewed together with MMN who served as an intermediary. After all reviewers agreed upon a final score for all items for all pilot CPGs, the 4 reviewers independently assessed each of the 7 eligible CPGs using the AGREE II. After review by the intermediary, reviewers were asked to re-evaluate items for which their scores differed by more than 2 points from the other reviewers. The intermediary provided no indication if scores were higher or lower than the other reviewers’ scores and did not require reviewers to change their scores.

#### Agree II

The AGREE II instrument consists of 23 items across 6 domains: (1) scope and purpose, (2) stakeholder involvement, (3) rigor of development, (4) clarity of presentation, (5) applicability, and (6) editorial independence [19]. A 24th item (overall guideline assessment) assesses a reviewer’s overall impression of a guideline. All items are rated on a 7-point Likert scale ranging from 1 (strongly disagree) to 7 (strongly agree). Domain scores range from 0% to 100% and enable scores to be compared across different domains because domains vary in the number of items they contain. Research on the AGREE II demonstrates that its domain scores have acceptable to good internal consistency (alpha range .64 to .89) and interrater reliability (alpha range .63 to .84) when 4 reviewers are used [28]. Importantly, the AGREE II is able to differentiate content designed to be of high and low quality [29]. For the purposes of this study, a domain cut-off score of 65% was used to determine guidelines of moderate to high quality. A cut-off score of 65% was chosen based on prior research identifying that guidelines approved by the Australian Government’s National Health and Medical Research Council typically achieve domain scores above 65% [30]. The AGREE II manual advises that domain scores should be interpreted within the context of a project; therefore, quality judgments were based also on inspection of an error bar graph.

#### Statistical Analysis

First, mean AGREE II item scores were calculated using the 4 reviewers’ item scores. Next, domain percentage scores were derived by summing all mean individual AGREE II item scores and standardizing the total as a percentage of the maximum possible score for that domain. Finally, the interrater reliability of the 4 reviewers was assessed using intraclass correlations (ICCs) for each guideline. A 2-way random model for absolute agreement was used. The ICCs were computed before and after arbitration by the intermediary.

### Study 2

#### Procedures

A convenience sample of health care professionals were recruited via Google advertisements restricted to Australia, as well as advertisements on the websites, newsletters, and email list servers of organizations whose members commonly provide substance use counseling. Advertisements specified that we were conducting a 30-minute online survey about cannabis use guidelines and that we were seeking health professionals who were involved in counseling individuals for cannabis use. The advertisements contained a hyperlink to the study information form. The information form specified that the purpose of the survey was to ascertain health professionals’ familiarity with guidelines for managing cannabis use and their opinions of them. The form also specified that if participants entered their email address at the end of the survey they would be entered into a draw to win 1 of 10 Aus $100 prizes via PayPal. Before initiating the online survey, participants were required to provide consent by clicking on the following option: “Yes, I have read the information and consent form and I am ready to participate.” Both the information form and survey were stored on the University of New South Wales website using Key Survey Enterprise. At the start of survey, individuals were asked whether or not they had counseled someone for cannabis use in the past 12 months. Only individuals who answered positively were allowed to continue. After 6 months of data collection (January to June 2012), the survey was closed and 10 participants were randomly chosen for the $100 prizes using a random number generator. At this point, data were downloaded from Key Survey Enterprise and stored in an intranet folder that only University of New South Wales study authors can access. Study 2 was approved by the Medical and Community Human Research Ethics Advisory Panel at the University of New South Wales.

#### Survey

The open survey contained demographic items that assessed participants’ gender, age, country of birth, educational background, profession, and practice area. Additional items assessed experience in counseling individuals who use cannabis and information sources participants used to inform their provision of cannabis use treatment. The remainder of the survey asked specific questions about the 7 CPGs identified during study 1 and used branch logic questioning in order to only ask questions which were relevant to the participant. For example, if a participant was not familiar with a particular CPG, no questions were asked about that guideline and the survey moved on to the next CPG. When a participant reported being familiar with a CPG, they were asked if they had read it and, if so, if they had used it. If participants had not read a CPG after encountering it, they were asked to report if it was because they were too busy, the CPG was too long, the CPG contained things they already knew, or if it was because the CPG conflicted with their theoretical orientation. When participants had read a CPG they were asked to rate the CPG on 9 Likert-scale items from 1 (strongly disagree) to 5 (strongly agree), where 3 represented neutral. Items were presented in the same order for each participant and were mandatory in that participants could not progress unless an item was answered. In addition, participants could only review items for the currently displayed items (ie, there was no “back” button). The usability and functioning of the survey was tested by the first and second authors before its public launch.

## Results

### Study 1

#### Clinical Practice Guideline Characteristics

Seven eligible guidelines met inclusion criteria (see Table 1). Two guidelines were specific to cannabis use: Management of Cannabis Use Disorder and Related Issues: A Clinician’s Guide published by the National Cannabis Prevention and Information Centre (NCPIC) [31] and Clinical Practice Guidelines for Management of Cannabis Dependence published by the Indian Psychiatric Society (IPS) [32]. The other CPGs, published by the National Institute for Health and Clinical Excellence (NICE), the American Psychiatric Association (APA), the New South Wales Department of Health, and New Zealand’s National Health Committee (NHC), were related to substance use treatment in general and only included sections specifically related to cannabis use. The New South Wales Department of Health published two guidelines: (1) Drug and Alcohol Psychosocial Interventions Professional Practice Guidelines [33] hereafter referred to as NSWD (New South Wales-Drug), and (2) National Clinical Guidelines for the Management of Drug Use During Pregnancy, Birth and the Early Development Years of the Newborn [34] hereafter referred to as NSWP (New South Wales-Pregnancy). The guidelines were developed in 5 different countries. Guideline length ranged from 12 to 338 pages.

**Table 1:** Clinical practice guidelines characteristics.

Clinical practice guideline^a^	Date of last update	Country of origin	Number of pages
Management of Cannabis Use Disorder and Related Issues: A Clinician’s Guide (NCPIC) [31]	2009	Australia	128
Clinical Practice Guidelines for Management of Cannabis Dependence (IPS) [32]	2006	India	12
Drug Misuse: Psychosocial Interventions (NICE) [35]	2008	United Kingdom	338
Practice Guideline for the Treatment of Patients with Substance Use Disorders (APA) [36]	2006	United States	276
Drug and Alcohol Psychosocial Interventions Professional Practice Guidelines (NSWD) [33]	2008	Australia	93
National Clinical Guidelines for the Management of Drug Use During Pregnancy, Birth and the Early Development Years of the Newborn (NSW) [34]	2006	Australia	116
Guidelines for Recognising, Assessing and Treating Alcohol and Cannabis Abuse in Primary Care (NHC) [37]	1999	New Zealand	36

^a^ NCPIC: National Cannabis Prevention and Information Centre; IPS: Indian Psychiatric Society; NICE: National Institute for Health and Clinical Excellence; APA: American Psychiatric Association; NSW: New South Wales Department of Health; and NHC: National Health Committee.

#### Interrater Reliability

Before arbitration, the ICCs ranged from 0.80 to 0.94. After the intermediary notified reviewers of scores that differed by more than 2 points from other reviewers, 50 of 672 item scores (7.4%) were changed. These changes led to ICCs between 0.89 and 0.96, demonstrating high interrater reliability.

#### Agree II

The 3 items that scored the lowest assessed whether the views of the target population had been sought, if the CPG had been externally reviewed by experts, and if the competing interests of the CPG development group had been recorded and addressed (see Table 2). The 3 highest scores were from the scope and purpose domain. These items assessed the quality of descriptions for the overall objective of the CPG, the health question(s) covered by the CPG, and the population for whom the CPG was intended. Across CPGs, average domain scores were 65% or greater in 2 instances: (1) scope and purpose and (2) clarity of presentation. The most variable domain scores across CPGs were for rigor of development (9% to 89%), followed by editorial independence (0% to 77%). These 2 domains also received the lowest mean domain scores, along with applicability.

**Table 2:** AGREE II mean item scores and domain percentage scores for each guideline.

Item		Guideline^a^							Overall mean score
		NCPIC	IPS	NICE	APA	NSWD	NSWP	NHC	
**Scope and purpose, mean item score**									
	Objective described	5.75	3.50	6.50	6.00	6.50	6.25	6.50	5.86
	Health question described	6.00	4.25	6.50	6.25	6.25	6.50	5.50	5.89
	Population described	6.00	4.50	6.50	5.75	5.75	6.50	6.25	5.89
	Domain score,^b^ %	82%	51%	92%	83%	86%	90%	85%	81%
**Stakeholder involvement, mean item score**									
	Relevant professional groups	5.25	2.25	6.50	3.75	5.00	5.75	5.00	4.79
	Target population preferences	2.75	1.00	5.50	1.25	1.75	1.75	3.75	2.54
	Target users defined	6.75	3.00	6.25	5.75	6.75	6.25	5.50	5.75
	Domain score,^b^ %	65%	18%	85%	43%	58%	60%	63%	56%
**Rigor of development, mean item score**									
	Systematic search	2.25	1.25	6.75	6.75	2.25	5.50	1.50	3.75
	Selection criteria described	3.75	1.50	6.75	6.25	2.25	4.50	4.25	4.18
	Strengths/limitations described	5.00	1.00	6.75	6.25	5.50	5.75	2.00	4.61
	Formulation methods described	5.25	1.50	6.75	6.00	5.50	3.75	1.75	4.36
	Risks/benefits considered	4.75	1.00	5.25	5.00	4.75	5.00	3.00	4.11
	Suggestions linked to evidence	5.50	3.50	6.75	6.50	6.25	6.25	4.00	5.54
	Externally reviewed by experts	1.00	1.00	5.00	5.00	1.25	1.75	1.25	2.32
	Procedure for updates	3.50	1.00	6.50	5.00	4.50	1.75	1.25	3.36
	Domain score,^b^ %	48%	9%	89%	81%	51%	55%	23%	50%
**Clarity of presentation, mean item score**									
	Specific recommendations	6.25	4.00	6.50	5.50	5.75	5.25	4.50	5.39
	Options presented	6.50	3.50	6.50	6.50	6.25	6.25	4.00	5.64
	Identifiable recommendations	6.25	4.00	4.50	3.25	6.50	4.75	6.25	5.07
	Domain score,^b^ %	89%	47%	81%	68%	86%	74%	65%	73%
**Applicability, mean item score**									
	Facilitators/barriers described	4.75	1.25	3.75	4.75	3.75	3.25	3.00	3.50
	Advice/tools for implementation	6.50	2.00	6.00	3.25	4.25	6.00	4.00	4.57
	Resource implications considered	3.25	1.00	6.75	4.00	3.00	3.00	1.50	3.21
	Monitoring criteria presented	4.00	2.00	5.25	4.25	4.00	4.25	3.50	3.89
	Domain score,^b^ %	60%	9%	74%	51%	46%	52%	33%	46%
**Editorial independence, mean item score**									
	Lack of funding body influence	4.25	1.00	4.50	3.50	3.75	3.50	1.50	3.14
	Competing interests addressed	1.25	1.00	6.75	5.25	1.00	1.00	1.00	2.46
	Domain score,^b^ %	29%	0%	77%	56%	23%	21%	4%	30%
**Overall guideline assessment, mean item score**									
	Overall quality	5.50	2.00	6.00	4.75	4.50	5.25	3.50	4.50

^a^ NCPIC: National Cannabis Prevention and Information Centre; IPS: Indian Psychiatric Society; NICE: National Institute for Health and Clinical Excellence; APA: American Psychiatric Association; NSWD: New South Wales-Drug; NSWP: New South Wales-Pregnancy; and NHC: National Health Committee.

^b^ Domain percentage scores were derived by summing all mean individual AGREE II item scores and standardizing the total as a percentage of the maximum possible score for that domain.

**Figure 2:**
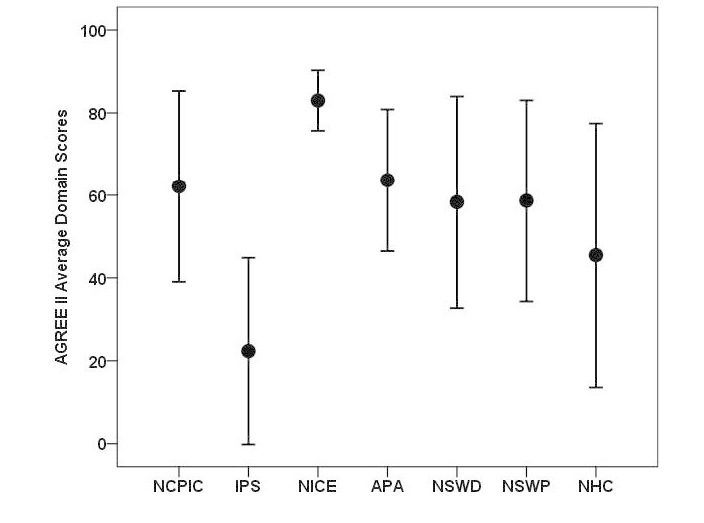
Mean AGREE II domain scores for the 7 eligible clinical practice guidelines. NCPIC: National Cannabis Prevention and Information Centre; IPS: Indian Psychiatric Society; NICE: National Institute for Health and Clinical Excellence; APA: American Psychiatric Association; NSWD: New South Wales-Drug; NSWP: New South Wales-Pregnancy; and NHC: National Health Committee.

The CPGs showed great variability across the domains, ranging from 0% to 92% adherence (Figure 2). The NICE guideline was the most consistent performing CPG and the only CPG to score above 65% in all domains (indicating high quality). This CPG also received the highest scores in all domains, except for clarity of presentation, which was obtained by the NCPIC guideline. The IPS guideline did not achieve over 65% on any domain and achieved the lowest scores across all domains compared to other CPGs (indicating very low quality). Guidelines other than the NICE and the IPS guidelines achieved average domain scores between 46% and 64% (indicating acceptable to low quality).

### Study 2

#### Participant Characteristics

A total of 190 individuals provided informed consent to participate in the study; however, only 166 of these individuals reported that they had counseled someone for cannabis use in the past 12 months, meeting the criteria to complete the survey. The average age of these 166 individuals was 45.47 years (SD 12.14). Of these, 64.5% (107/166) were female and 77.7% (129/166) were born in Australia. The next most common country of origin was England (7.2%, 12/166). Educational and professional details for the participants are presented in Table 3. Most participants had received some type of tertiary education, worked for an organization, practiced in a metropolitan area, were a drug and alcohol worker/specialist, and had over 3 years’ experience counseling individuals who use cannabis (Table 3). Slightly more of the study sample reported being from regional or remote areas compared to the entire Australian population [38]. Guidelines, websites, colleagues, and workshops were the most frequently identified sources for informing participants’ treatment of cannabis use.

**Table 3:** Educational and professional characteristics of the participants (N = 166).

Characteristics		n (%)
**Educational background**		
	No higher education degree	4 (2.4%)
	Certificate/diploma/advanced diploma	53 (31.9%)
	Bachelor’s degree	62 (37.3%)
	Master’s degree	34 (20.5%)
	MD/PhD or equivalent	13 (7.8%)
**Employment status**		
	Employee of organization	150 (90.4%)
	Self-employed	15 (9.0%)
	Unemployed	1 (0.01%)
**Practice area**		
	Metropolitan	106 (63.9%)
	Rural	54 (32.5%)
	Remote	6 (3.6%)
**Profession**		
	Drug and alcohol worker/specialist	71 (42.7%)
	Counselor	10 (6.0%)
	Social worker	10 (6.0%)
	Psychologist	32 (19.3%)
	Psychiatrist	2 (1.2%)
	Nurse	24 (14.5%)
	Other	17 (10.2%)
**Cannabis counseling experience**		
	0-6 months	8 (4.8%)
	6-11 months	12 (7.2%)
	1-3 years	35 (21.1%)
	3-5 years	26 (15.7%)
	Over 5 years	85 (151.2%)
**Cannabis use treatment information sources** ^**a**^		
	Guidelines	103 (62.0%)
	Websites	96 (57.8%)
	Journal articles	100 (60.2%)
	Books	69 (41.6%)
	Workshops	100 (60.2%)
	Conferences	67 (40.4%)
	Colleagues	99 (59.6%)

^a^ Participants were able to choose more than one option, thus, percentages do not sum to 100.

#### Familiarity with Clinical Practice Guidelines

Participants were most familiar with the NCPIC, NSWD, and NICE guidelines; however, less than half of the study participants (44.6%, 74/166) were aware of the NCPIC guideline, the most frequently encountered CPG (Table 4). Peers/colleagues and websites were the top reasons cited for knowing about all 3 of these CPGs. Professional development workshops also were key in disseminating the NCPIC guideline, as were journal articles for the NICE guideline, and senior professionals for the NSWD guideline. The 3 least frequently encountered guidelines (APA, NSWP, and NHC) also were often discovered via these routes with the addition of education programs.

In most cases, approximately three-fourths of individuals had read the CPGs they had encountered; however, over 90% of individuals had read the NSWD and NSWP guidelines after they heard about them. These 2 CPGs were encountered through senior professionals more so than the other guidelines. The most common reasons acknowledged for not reading a CPG was being too busy (40% to 83% of respondents for all CPGs), believing a CPG was at conflict with one’s theoretical orientation (0% to 50% for all CPGs), believing a CPG was too long (0% to 40% for all CPGs), and already knowing the content (0% to 33% for all CPGs). In many cases, after a participant had read a CPG they were likely to use it in their practice. This was most often the case for the NCPIC and APA guidelines. Both of these CPGs were encountered through workshops more so than any other guideline.

Examination of mean scores demonstrates that participants tended to agree or feel neutral toward the quality statements about the selected CPGs (Table 5). Thus, little variability in item scores existed between the CPGs. For 6 of the 9 items, participants scored the NSWP guideline slightly higher. In regards to the 3 other items, participants reported that the NSWD guideline was the easiest to follow, the NCPIC guideline was the most clearly presented, and the NCPIC and NSWD guidelines were the most applicable to their practice. On average, the NHC guideline scored slightly lower across 6 items. In 2 cases, the NICE guideline was rated the lowest. Participants were less supportive of routinely using the NICE guideline and in less agreement that they were easy to follow compared to the other CPGs. Lastly, participants reported that the NCPIC guideline was based on patient preferences the least.

**Table 4:** Familiarity with clinical practice guidelines (CPGs).

Item		Guideline^a^						
		NCPIC n (%)	IPS n (%)	NICE n (%)	APA n (%)	NSWD n (%)	NSWP n (%)	NHC n (%)
Familiar with the CPG		74 (44.6%)	35 (21.1%)	49 (29.5%)	32 (19.3%)	55 (33.1%)	30 (18.1%)	8 (4.8%)
**CPG discovery method** ^**b**^								
	Education program	7 (10%)	3 (9%)	7 (14%)	6 (19%)	9 (16%)	8 (27%)	3 (38%)
	Journal article	6 (8%)	4 (11%)	9 (18%)	5 (16%)	5 (9%)	2 (7%)	1 (13%)
	Peers/colleague	26 (35%)	11 (31%)	11 (22%)	12 (38%)	18 (33%)	10 (33%)	2 (25%)
	Senior professional	10 (14%)	5 (14%)	8 (16%)	14 (4%)	13 (24%)	7 (23%)	0 (0%)
	Mailing list	13 (18%)	2 (6%)	8 (16%)	1 (3%)	7 (13%)	4 (13%)	1 (13%)
	Conference	9 (12%)	3 (9%)	6 (12%)	3 (9%)	3 (6%)	3 (10%)	1 (13%)
	Workshop	17 (23%)	6 (17%)	4 (8%)	6 (19%)	2 (4%)	1 (3%)	1 (13%)
	Website	16 (22%)	14 (40%)	18 (37%)	7 (22%)	17 (31%)	10 (33%)	3 (38%)
Read the CPG^b^		55 (74%)	29 (83%)	37 (76%)	24 (75%)	50 (91%)	28 (93%)	6 (75%)
Used the CPG after reading^c^		55 (100%)	24 (83%)	33 (89%)	23 (96%)	45 (90%)	24 (86%)	5 (83%)

^a^ NCPIC: National Cannabis Prevention and Information Centre; IPS: Indian Psychiatric Society; NICE: National Institute for Health and Clinical Excellence; APA: American Psychiatric Association; NSWD: New South Wales-Drug; NSWP: New South Wales-Pregnancy; and NHC: National Health Committee.

^b^ Percentages were calculated based on how many people were familiar with that particular guideline and not the total sample.

^c^ The denominator for calculating the percentage is equal to the guideline n of Read the CPG.

**Table 5:** Treatment providers’ opinions regarding the clinical practice guidelines.

Item	Guideline,^a^ mean (SD)						
	NCPIC	IPS	NICE	APA	NSWD	NSWP	NHC
Should be routinely used	3.70 (0.68)	3.49 (0.61)	3.42 (0.67)	3.44 (0.76)	3.67 (0.82)	3.83 (0.70)	3.50 (0.53)
Easy to follow	3.78 (0.60)	3.63 (0.49)	3.62 (0.57)	3.66 (0.65)	3.87 (0.67)	3.80 (0.61)	3.63 (0.74)
Clear who should use	3.80 (0.72)	3.69 (0.58)	3.60 (0.53)	3.56 (0.67)	3.82 (0.67)	3.90 (0.71)	3.38 (0.52)
Based on patient preferences	3.03 (0.70)	3.14 (0.60)	3.12 (0.56)	3.16 (0.57)	3.24 (0.69)	3.30 (0.84)	3.25 (0.46)
Clearly presented	3.91 (0.58)	3.74 (0.51)	3.62 (0.60)	3.60 (0.71)	3.89 (0.79)	3.80 (0.61)	3.50 (0.53)
Applicable to my practice	3.91 (0.69)	3.77 (0.60)	3.64 (0.60)	3.75 (0.62)	3.91 (0.80)	3.87 (0.82)	3.63 (0.74)
Rigorously developed	3.66 (0.71)	3.69 (0.68)	3.52 (0.54)	3.66 (0.71)	3.60 (0.78)	3.77 (0.63)	3.25 (0.46)
Would recommend	3.88 (0.64)	3.80 (0.68)	3.52 (0.58)	3.63 (0.71)	3.85 (0.78)	4.00 (0.64)	3.50 (0.93)
Overall quality is good	3.95 (0.62)	3.83 (0.62)	3.64 (0.56)	3.72 (0.63)	3.80 (0.68)	3.97 (0.67)	3.63 (0.74)

^a^ NCPIC: National Cannabis Prevention and Information Centre; IPS: Indian Psychiatric Society; NICE: National Institute for Health and Clinical Excellence; APA: American Psychiatric Association; NSWD: New South Wales-Drug; NSWP: New South Wales-Pregnancy; and NHC: National Health Committee.

## Discussion

Publishing CPGs online is intended to facilitate the dissemination of evidence-based treatment information; however, the provision of these resources through the Internet alone does not guarantee practitioner uptake [13-15]. This caveat combined with the lack of quality control associated with online resources necessitated an evaluation of treatment providers’ access to and options of CPGs for managing cannabis use, as well as an evaluation of their scientific soundness. Utilizing 4 trained CPG reviewers, study 1 found a high amount of variability between CPGs for managing cannabis use, whereas treatment providers in study 2 reported much less variability between the CPGs. In addition, these studies identified areas of improvement for guideline developers and a potential dissemination combination that may lead to greater CPG familiarity and implementation.

### Clinical Practice Guideline Quality

Based on AGREE II domain scores and examination of an error bar graph, the CPGs fell into 3 broad categories: high quality, acceptable to low quality, and very low quality. The NICE guidelines had the highest overall quality; the NCPIC, APA, NSWD, NHC, and NSWP guidelines were deemed acceptable to low quality; and the IPS guidelines were rated as needing substantial improvement. Inspection of domain scores demonstrated that consistent with prior research, CPGs performed the poorest in the areas of rigor of development, applicability, and editorial independence [8-10]. As such, CPG developers can enhance the quality of revisions to these CPGs by substantially improving reporting in these 3 areas.

Interestingly, findings from studies 1 and 2 were somewhat incongruent. In general, treatment providers assessed during study 2 reported substantially less variability in CPG quality as compared with the reviewers from study 1. All 9 areas of interest examined during study 2 received average scores. Although the clinical significance between mean item scores is likely low, examination of these scores further highlights the quality discrepancies reported by treatment providers and trained reviewers. For example, 3 of the 9 items assessed during study 2 are directly comparable to AGREE II domain scores (rigor of development, clarity of presentation, and applicability). In all of these areas, the trained reviewers rated the IPS guideline the lowest, whereas the treatment providers rated the NHC guideline the lowest. Treatment providers rated the IPS guideline second- to fourth-best in these areas. Examination of study 2 items that were similar to AGREE II items (defining target users, seeking patient preferences, and overall quality) demonstrated a similar discrepancy. In all cases, the IPS guideline was rated the lowest by the trained reviewers, whereas treatment providers reported that the NHC guideline was the poorest in terms of clarity about who should use the guideline and overall quality. Treatment providers also reported that the NCPIC guideline was based less on patient preferences than the other guidelines. The discrepancies in ratings between reviewers and treatment providers indicate that treatment providers may not be able to differentiate between good and poor CPG reporting quality. This finding is important because it may suggest that treatment providers are at risk for adopting CPGs that are not based on the best available evidence.

Although validity testing has demonstrated that the AGREE II is able to differentiate higher quality reporting from lower quality reporting [29], it is possible that well-reported CPGs contain flawed recommendations and poorly reported CPGs contain sound recommendations. A comparison of reports and research protocols for randomized controlled trials conducted by the Radiation Therapy Oncology Group showed that the methodological quality of studies was often substantially better than that reported [39]. For example, allocation concealment and sample size calculations were only reported in 42% and 16% of the reports, but reported in 100% and 76% of the research protocols. If these results are transferable to CPGs, it may mean that the scientific soundness of the poorest performing CPGs according to AGREE II ratings may be substantially better than thought. It is important to note, however, that all research protocols produced by the Radiation Therapy Oncology Group must pass through a rigorous peer review process and be approved through its own committee system and the National Cancer Institute before a randomized controlled trial can progress. Thus, CPGs that do not undergo peer review during development or afterwards may not be better than what AGREE II scores suggest.

### Dissemination and Impact

Study 2 demonstrates that the effectiveness of current CPG dissemination methods is suboptimal. Slightly less than half of the study population was familiar with the NCPIC guideline, the most well-known guideline, whereas only a third of the sample had heard of the second most commonly identified guideline (NSWD). Prior research has found that 59% to 98% of substance abuse treatment providers are familiar with motivational and cognitive-behavioral treatment approaches [40-42]. Because these approaches have the most evidence base for treating cannabis use, unless CPGs reach the 49% of providers who do not have preexisting knowledge about these evidence-based approaches, current CPG access rates may not lead to an increase in standards of care. Examination of potential differences in dissemination efforts between the NCPIC guidelines and the other 6 CPGs may suggest methods for improvement because the NCPIC guideline was the most commonly encountered.

Peers/colleagues, websites, and workshops were the most common methods reported for discovery of the NCPIC guidelines. As peers/colleagues and websites were common methods for discovering all identified CPGs, the addition of workshop dissemination may lead to increased CPG familiarity. Previous research suggests that greater adoption of workshop materials is facilitated by greater relevancy of training (eg, information obtained is relevant to the needs of participants’ clients) and greater program support (eg, having enough time to implement the materials) [43]. As such, dissemination via workshops may only increase cannabis-related CPG uptake for participants who regularly provide psychosocial treatment for cannabis treatment and who have the time and support of their organization for CPG implementation. In support of this assumption, the NSWD and NSWP guidelines were the most frequently read once encountered, and were heard of more often via senior professionals than the other guidelines. CPG access through senior professionals may be a proxy indicator of organizational support. In addition, the NCPIC and APA guidelines were the most frequently used once read, and these CPGs were accessed via workshops more than the other guidelines. Based on these combined findings, successful dissemination and implementation may be facilitated by the combination of peers/colleagues, senior professionals, workshops, and websites.

To increase the uptake of guideline usage, CPGs should further take into account the needs of the treatment provider. Consistent with previous research on evidence-based treatment and CPG adoption [29,40,43], barriers commonly reported during study 2 for not reading a CPG included a lack of time, CPG length, conflict with theoretical orientation, and already being familiar with the content. Only a lack of time and CPG length can be addressed by guideline developers. Accordingly, finding ways to make CPGs more time efficient while not compromising on quality should be a primary objective of future guideline development. A method for achieving this goal may be for CPG developers to publish two documents online. One document could focus on the content of practice, whereas the other could be reserved for reporting development processes and conflicts of interest.

### Strengths and Limitations

The current research has several strengths, including a systematic evaluation of CPGs conducted by multiple reviewers with a high level of consistency among the reviewers. Additionally, the evaluation was conducted using a psychometrically robust assessment tool. Finally, the systematic evaluation study was followed with data from users of the guidelines, enabling us to examine gaps between quality and real-world perceptions of the guidelines. These strengths must be considered in light of study limitations. First, the AGREE II instrument provided an indication of guideline reporting quality, rather than a direct indication of the appropriateness of the recommendations. Previous research cautions that cannabis use information for patients available online is not of a high standard [44]. Therefore, an important follow-up study would involve a content assessment of the identified CPGs. Second, study 2 did not provide a direct comparison of guidelines because this would have required every treatment provider to read and evaluate each CPG. This was not feasible, especially since this study identified that treatment providers did not have enough time to read the CPGs that they had heard of. Third, the type of data obtained through studies 1 and 2 prevented the use of inferential analyses for examining associations between the studies’ findings (eg, 4 data points for each AGREE II item because of 4 reviewers and a highly varied sample size for treatment provider opinions based on differential CPG familiarity rates). Lastly, the health provider survey we developed for this study utilized a 5-point response format. This response scale limited the variability of providers’ ratings and also prevented direct comparison with the AGREE II.

### Conclusions

This research provided the first evaluation of online CPGs that address psychosocial treatments for reducing cannabis use. The findings provide an indication of the reporting quality of CPGs that are freely available to treatment providers, and highlight gaps between the quality of CPGs as assessed by a psychometrically validated assessment tool and treatment provider perceptions. The research also suggests possible methods for increasing the uptake of CPGs among treatment providers.

## Abbreviations

ADCA: Alcohol and Other Drugs Council of Australia
AGREE II: Appraisal of Guidelines for Research and Evaluation
APA: American Psychiatric Association
CPG: clinical practice guideline
ICC: intraclass correlation
IPS: Indian Psychiatric Society
NCPIC: National Cannabis Prevention and Information Centre
NDSIS: National Drugs Sector Information Service
NHC: National Health Committee
NICE: National Institute for Health and Clinical Excellence
NSWD: New South Wales Department of Health (Drug and Alcohol Psychosocial Interventions Professional Practice Guideline)
NSWP: New South Wales Department of Health (National Clinical Guidelines for the Management of Drug Use During Pregnancy, Birth and the Early Development Years of the Newborn guideline)
